# Artifacts In Magnetic Resonance Imaging and Computed Tomography Caused By Dental Materials

**DOI:** 10.1371/journal.pone.0031766

**Published:** 2012-02-22

**Authors:** Thomas Klinke, Amro Daboul, Juliane Maron, Tomasz Gredes, Ralf Puls, Ahmad Jaghsi, Reiner Biffar

**Affiliations:** 1 Polyclinic of Prosthodontics and Biomaterials, Greifswald University. Greifswald, Germany; 2 Polyclinic of Orthodontics, Greifswald University. Greifswald, Germany; 3 Institute of Diagnostics, Radiology and Neuroradiology, Greifswald University, Greifswald, Germany; National Institute of Health, United States of America

## Abstract

**Background:**

Artifacts caused by dental restorations, such as dental crowns, dental fillings and orthodontic appliances, are a common problem in MRI and CT scans of the head and neck. The aim of this in-vitro study was to identify and evaluate the artifacts produced by different dental restoration materials in CT and MRI images.

**Methods:**

Test samples of 44 materials (Metal and Non-Metal) commonly used in dental restorations were fabricated and embedded with reference specimens in gelatin moulds. MRI imaging of 1.5T and CT scan were performed on the samples and evaluated in two dimensions. Artifact size and distortions were measured using a digital image analysis software.

**Results:**

In MRI, 13 out of 44 materials produced artifacts, while in CT 41 out of 44 materials showed artifacts. Artifacts produced in both MRI and CT images were categorized according to the size of the artifact.

**Significance:**

Metal based restoration materials had strong influence on CT and less artifacts in MRI images. Rare earth elements such as Ytterbium trifluoride found in composites caused artifacts in both MRI and CT. Recognizing these findings would help dental materials manufacturers and developers to produce materials which can cause less artifacts in MRI and CT images.

## Introduction

Today, various kinds of materials are used in the dental treatment, materials such as metal alloys, composites, acrylics, porcelain and ceramics are used as filling materials and in dental prosthesis like crowns, dental bridges and dentures. The properties and specifications of these materials are well defined and studied. However, their influence and effect on image quality with computed tomography (CT) and magnetic resonance imaging (MRI) is not covered sufficiently in the literature.

In CT imaging, Streak artifacts are a common problem. The presence of high attenuation metal objects in the field of view such as dental restorations, orthodontic bands, surgical plates and pins can cause this type of artifacts. That is because the metal materials highly attenuate the x-ray beam resulting in incorrect high attenuation values of objects behind the metal. However, in MRI, images are created using a combination of strong uniform magnetic field and radio frequency pulses. All substances when placed in a magnetic field are magnetized at various degrees depending on their magnetic susceptibility. The variations in the magnetic field strength that occur on the interface between the dental material and the adjacent tissues will cause magnetic field distortions and signal loss which will generate an artifact in the image. The artifact severity will vary depending on the shape, position, orientation and number of objects in the image, sequence type used and sequence parameters [1]–[5].

MRI and CT image quality can be dramatically degraded by artifacts caused by dental materials, which limits their usefulness as diagnostic tools. Artifacts might obscure a pathology (e.g. Tumors, Inflammatory tissues) or obscure the anatomy of the area examined and make it difficult to locate anatomical structure for surgical procedures [2], [6].

Many studies have investigated the artifacts generated by metals used in medicine and dentistry on MRI and CT [1]–[5], [7]–[17]. However, the effect of non-metal based materials on MRI and CT image quality was not covered sufficiently. Hinshaw et al. [13] discussed artifacts that were caused by some materials commonly used in dental restorations, mainly stainless steel materials, such as orthodontic bands, braces, metal pins and posts.Fache et al. [11] evaluated a variety of dental materials and the mechanism of artifact production in MRI. Their study analyzed the composition and magnetic susceptibility of tested dental materials and compared the results with the artifacts produced by each material. It was concluded that the size of an artifact is related to the magnetic permeability of the dental material. New et al. [18] investigated the deflection force of clips and the intensity of dental amalgam in MRI artifacts. Starcukova et al. [19] showed that MR imaging without artifacts is possible even close to dental devices (amalgam, precious alloys and titanium) only if they are made of materials with low magnetic susceptibility. However, they mentioned that not all dental materials in the current use meet this criterion of low magnetic susceptibility.

Although the previously mentioned studies have described the effects of metallic objects on MRI interpretation, few have addressed the effect of non-metal based materials on MRI or CT image quality.

The purpose of this study was to identify and evaluate the artifacts produced by different dental materials (Metal and Non-Metal) in CT and MRI images.

## Materials and Methods

**Figure 1:**
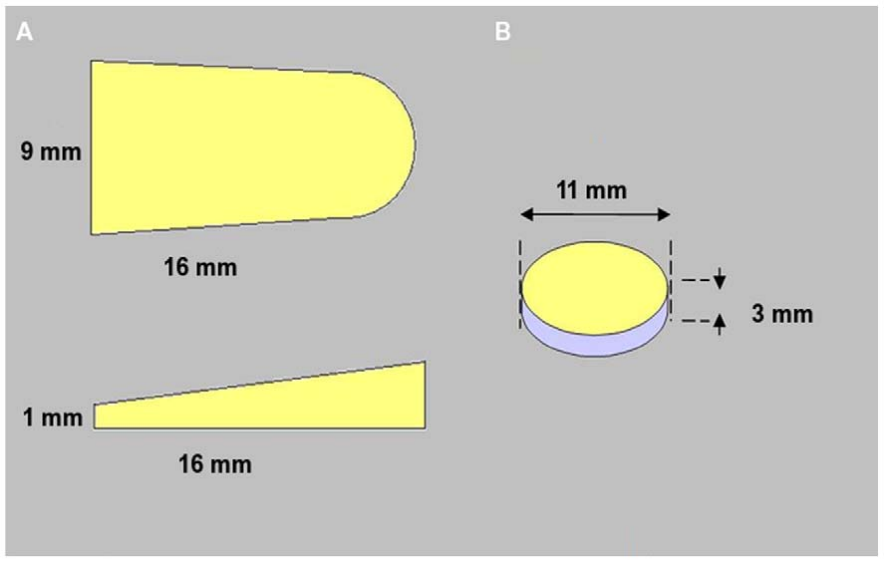
Dimensions of (A) wedge-shaped specimens and (B) reference.

Samples of 44 different materials (metal and non-metal), which are commonly used in dental practice, were included in this study (N = 44). The samples were divided into 4 groups; group 1: Composites (N = 31), group 2: temporary filling materials (N = 4), group 3: Dental ceramics (N = 5) and group 4: Metal alloys (N = 4). The test specimens were wedge-shaped with the dimensions of 16.0 mm in length by 9.0 mm in width, 3.5 mm high at the rear and 1 mm high at the tip (Figure 1a). Additionally, acrylic round reference specimens with an 11.0 mm diameter by 3.0 mm height were made (Figure 1b). We selected the wedge shape to have different material thickness from the tip and rear of each wedge, which will allow comparing the effect of artifacts produced by the test specimens of varying thickness on the adjacent acrylic round reference specimens.

Both, test specimens and reference specimens were embedded in gelatin (RUF Lebensmittelwerk KG, Quakenbrueck, Germany). The gelatin was mixed with water, cooked at 80°C for 2 minutes, poured in a mould about 60 mm from the base. After the first layer of gelatin had gelled, the tested samples and reference samples were placed in the middle of the mould. A second layer of gelatin was poured to entirely fill the mould. The embedded samples were placed in a CT (Sensation 16, Siemens, Erlangen Germany) and 1.5T MRI (Symphony 1.5T, Siemens, Erlangen Germany) respectively.

A spiral CT technique was used with 4 mm thickness, the parameters used were: 250 mA, 120 kV, 0.75 collimation and re-constructive thickness of 1 mm.

In the MRI, images were taken in the axial and sagittal planes. A T1-weighted spin-echo sequence (TR: 650 ms, TE: 40 ms, TSE bandwidth: 139 Hz/Pixel) and T2-weighted turbo spin-echo sequence (TR: 3000 ms, TE: 90 ms, TSE bandwidth: 130 Hz/Pixel). Images were taken with the following parameters: thickness 3.0 mm, matrix size 512×512 pixel; field of view (FOV) 250×250 mm.

Area of interest (AOI) was selected, artifact size and distortions in MRI were measured using Dicom image processing software (Osirix v 3.8 32bit). Lengths of artifacts produced in the CT were measured using an analyzing software (GE Advantage Workstation AW 4.207). In both softwares, all measurements were performed with the help of multi-planar reconstruction technique (MPR).

## Results

In both MRI and CT, the thickness of the wedge-shaped samples had no significant influence on artifacts produced.

Artifacts produced in MRI and CT were categorized according to the size and direction of the artifact. the artifact categories (Table 1) were in a similar manner to the classifications of Hinshaw et al. [13] and Behr et al. [4]. Artifacts produced by each group of materials were categorized: Composites (Table 2), Temporary filling materials (Table 3), Ceramics (Table 4) and Metal alloys (Table 5).

**Table 1:** MRI and CT artifact categories.

Category	0	1	2	3
MRI	No artifacts	Artifact less than 30.0 mm^2^	Artifact 30.0 mm^2^ to 60.0 mm^2^	Artifact more than 60.0 mm^2^
CT	No artifacts	Streak artifact <15.0 mm	Streak artifact 15.0–30.0 mm	Streak artifact >30.0 mm

**Table 2:** MRI and CT artifacts produced by composites (Group 1).

Material	CT artifact category	MRI T1 artifact category	MRI T1 artifact Shape change	MRI T2 artifact category	MRI T2 artifact Shape change
Adaptic®	1	0		0	
X-flow™	1	0		0	
Quixfil™	1	0		0	
Core X™	2	0		0	
Dyract® Xtra	2	0		0	
Ceram·X Duo	2	0		0	
Esthet•X®	3	0		0	
Dyract® flow	2	0		0	
Spectrum®TPH®	3	0		0	
X-tra fil	2	0		0	
Grandio	1	0		0	
Admira	3	0		0	
Twinky Star	2	0		0	
Arabesk®	2	0		0	
Tetric EvoCeram®	3	1	−/+−	1	−/(+)
Tetric Evoflow®	3	1	−/+−	1	−/(+)
Adamant®	3	1	−/+−	1	−/(+)
InTen-S®	2	1	−/+−	1	−/(+)
Tetric® Flow	3	1	−/+−	1	−/+
Tetric Ceram®	3	1	−/+−	1	−/(+)
Compoglass® F	3	1	−/+−	1	−/(+)
Heliomolar®	2	1	−/+−	1	−/(+)
Helio Progress®	0	0		0	
Filtek™	1	0		0	
3 M™ Z100™ MP	1	0		0	
Nanosit™	3	0		0	
Synergy D6	1	0		0	
Solidex	0	0		0	
Henry Schein®	2	0		0	
Charisma®	2	0		0	
Revolcin® Flow	1	0		0	

−/+ Test specimen not recognizable, adjacent reference specimen recognizable.

−/+− Test specimen not recognizable, adjacent reference specimen partially recognizable.

−/(+) Test specimen not recognizable, adjacent reference specimen recognizable but changed size or shape.

**Table 3:** MRI and CT artifacts produced by temporary filling materials (Group 2).

Material	CT artifact category	MRI T1 artifact category	MRI T1 artifact Shape change	MRI T2 artifact category	MRI T2 artifact Shape change
Clip	0	0		0	
Guttapercha	2	0		0	
Ketac™	2	0		0	
Cavit™ G	2	1	−/+−	1	−/+

−/+ test specimen not recognizable, adjacent reference specimen recognizable.

−/+− test specimen not recognizable, adjacent reference specimen partially recognizable.

**Table 4:** MRI and CT artifacts produced by Cermics (Group 3).

Material	CT artifact category	MRI T1 artifact category	MRI T1 artifact Shape change	MRI T2 artifact category	MRI T2 artifact Shape change
Zirconium dioxide	3	0		0	
IPS Empress®	1	0		0	
Vita Omega 900	2	0		0	
Cergo	2	0		0	
Duceragold	1	0		0	

**Table 5:** MRI and CT artifacts produced by Metal alloys (Group 4).

Material	CT artifact category	MRI T1 artifact category	MRI T1 artifact Shape change	MRI T2 artifact category	MRI T2 artifact Shape change
Amalcap® Plus	3	1	−/+	1	−/+
Remanium® Star	3	1	−/+	1	−/+
Degunorm®	3	1	−/+	1	−/+
Kavo Everest® Titan	3	1	−/+−	1	−/+−

−/+ test specimen not recognizable, adjacent reference specimen recognizable.

−/+− test specimen not recognizable, adjacent reference specimen partially recognizable.

**Figure 2:**
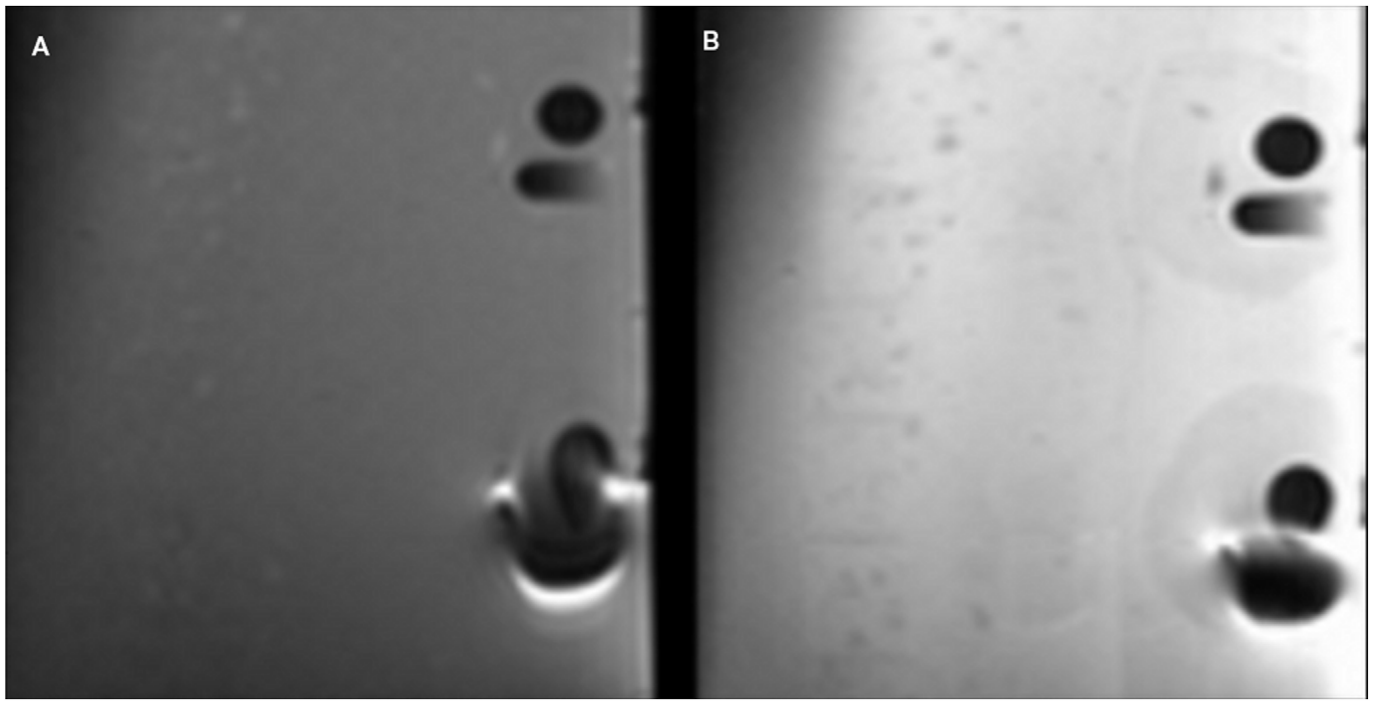
MRI artifact produced by Tetric EvoCeram in (A) T1 and (B) T2.

**Figure 3:**
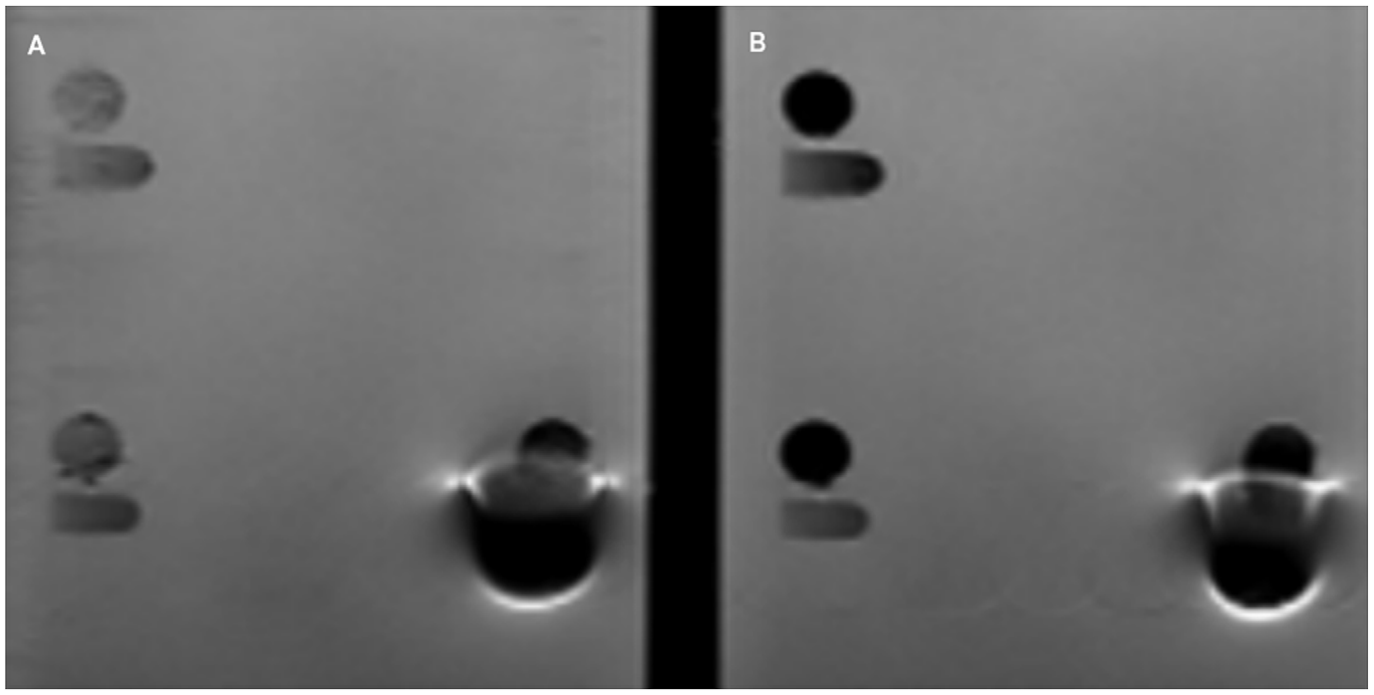
MRI artifact produced by Remanium in (A) T1 and (B) T2.

In MR images, there were no significant differences in artifact area extent between T1 and T2. In group 1 (Composites) 23 materials showed no artifacts (category 0), while 8 materials were assigned to category 1 (Figure 2). In group 2 (Temporary filling materials), 3 materials showed no artifacts, while only one material (Cavit) was classified in category 1. In group 3 (Ceramics) all 5 materials showed no artifacts. In group 4 (metal alloys) 4 materials were classified in category 1, while 2 materials (Remanium Star and Degunorm) showed object projections in an area far from the object itself(Figure 3).

**Figure 4:**
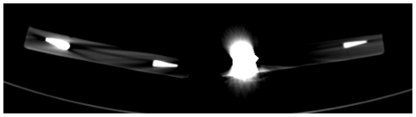
Streak artifact produced by Zirconium dioxide in CT.

**Figure 5:**
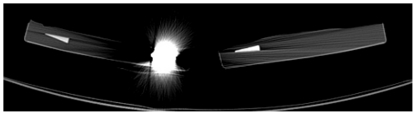
Streak artifact produced by Amalcap in CT.

In CT, group 1 (Composites) 2 materials (Helio Progress and Solidex) showed no streak artifacts (Category 1), 8 materials showed artifacts less than 15.0 mm (Category 2), 11 materials showed streak artifacts between 15.0 mm to 30.0 mm (Category 3) and 10 materials showed artifacts larger than 30.0 mm (Category 4). In group 2 (Temporary filling materials) 1 material (Clip) showed no artifacts (category 1) and 3 materials showed streak artifacts between 15.0 mm to 30.0 mm (Group 3). In group 3 (Ceramics), 2 materials (IPS Empress and Duceragold) showed artifacts less than 15.0 mm (Category 2), 2 materials (Cergo and Vita Omega 900) showed artifacts between 15.0 mm to 30.0 mm (Category 3) and 1 material (Zirconium dioxide) showed artifacts larger than 30.0 mm (Category 4) (Figure 4). In group 4 (Metal alloys), all 4 materials showed artifacts larger than 30.0 mm (Category 4)(Figure 5).

## Discussion

Various metal restorations and prosthesis may limit the usefulness of CT and MRI imaging, either by degrading the quality of the image or causing disturbances in the image, both of which will complicate the image interpretation and subsequently the diagnosis.

In CT, metal based materials can cause severe artifacts [20], because attenuation data of the CT scan are distorted by the high density of metallic objects, leading to inconsistencies which prevents adequate calculation of the projection data, commonly resulting in a star burst artifact which consists of radiating lines originating from the metal alloy.

In MRI, the presence of ferromagnetic metals in some of the dental materials causes magnetic field inhomogeneity [21], where metal-based materials create their own magnetic field and dramatically alter precession frequencies of protons in the adjacent tissues. Tissues adjacent to ferromagnetic components become influenced by the induced magnetic field of the metal, therefore, they either fail to precess or do so at a different frequency, hence they do not generate a useful signal. However, in this study, it was shown that not only metal based dental materials causes susceptibility artifacts, but also Non-Metal materials can cause artifacts and disturbances in the CT and MRI images.

The ceramic frame material (Zirconium dioxide) led surprisingly to the same effect of metal-based materials and disturbed the delineation of anatomic structures in the CT images. Furthermore, Ingredients like Ytterbium trifluoride, Ferric oxide and Lanthanum oxide, which can be found in composites as coloring agents caused image disturbances in CT and MRI.

In MRI, this is due to the fact that these materials contain some ferromagnetic metal ingredients. According to Eggers et al. [10], even small amounts of a ferromagnetic substance can cause an extensive signal void in the image.

Diamagnetic materials such as gold and paramagnetic materials like titanium are used extensively in oral restorations and are less likely to create artifacts in MRI. However, in this study they generated artifacts and distortions in both MRI and CT images. That is because the alloys contained traces of others ferromagnetic metals such as iron. Furthermore, paramagnetic materials that are used as additives in dental materials and prosthetic appliances could cause artifacts. In a study done by Bartels et al. [22] paramagnetic substances in vascular stents caused artifacts on MRI, the authors explained that the generated artifacts depended on the MRI sequence used and the material size and thickness. Camacho et al [23] investigated MRI artifacts caused by radiofrequency eddy currents. They concluded that the resulted substantial signal intensity artifact, in addition to any susceptibility effect, also depends on the shape, orientation, and material of the object under investigation.

As mentioned earlier, the magnitude of susceptibility artifacts in MRI is also related to the type of imaging sequence used. Gradient echo (GRE) sequences are sensitive to the presence of metal, where intravoxel dephasing is the predominant cause of signal loss, resulting in a dark or black area around the metal on the processed images. [24] Shortening the echo time (TE) and decreasing voxel size can be used to reduce the degree of intravoxel dephasing seen on GRE acquisition. [25] On the other hand, Spin-echo (SE) sequences have a 180° RF-pulse that refocuses the spins at the echo time and thereby diminishes the phase shifts in the voxel which are caused by local static magnetic field gradients. The refocusing 180° RF-pulse makes the SE sequence less sensitive to susceptibility effects [26].

In this study, we did not try artifact reduction with short TE, and the sequence protocol in both scans (T1 with TE 40 ms, T2 with TE 90 ms) showed similar artifact sizes and shapes for most of the material tested.

Considering the results of this study, the use of ceramic materials like Zirconium dioxide in crowns, bridges or permanent fillings has to be critically analyzed in the case of CT or MR imaging of the lower mid face.

Further more, dental materials developers would benefit from the results of this study to produce materials that cause less artifacts and distortions in CT and MRI images.
